# SARS-CoV-2-specific CD4^+^ T cells are associated with long-term persistence of neutralizing antibodies

**DOI:** 10.1038/s41392-022-00978-0

**Published:** 2022-04-23

**Authors:** Zhongfang Wang, Xiaoyun Yang, Xinyue Mei, Yumin Zhou, Zhiqiang Tang, Guichang Li, Jiaying Zhong, Mengqiu Yu, Mingzhu Huang, Xiaoling Su, Bijia Lin, Pengxing Cao, Ji Yang, Pixin Ran

**Affiliations:** 1 State Key Laboratory of Respiratory Disease & National Clinical Research Center for Respiratory Disease, Guangzhou Institute of Respiratory Health, The First Affiliated Hospital of Guangzhou Medical University, Guangzhou Medical University, Guangzhou, China; 2 The Second People’s Hospital of Changde, Hunan, China; 3 School of mathematics and Statistics, University of Melbourne, Melbourne, VIC Australia

**Keywords:** Adaptive immunity, Infectious diseases, Lymphocytes

## Abstract

Understanding the decay and maintenance of long-term SARS-CoV-2 neutralizing antibodies in infected or vaccinated people and how vaccines protect against other SARS-CoV-2 variants is critical for assessing public vaccination plans. Here, we measured different plasm antibody levels 2 and 12 months after disease onset, including anti-RBD, anti-N, total neutralizing antibodies, and two neutralizing-antibody clusters. We found that total neutralizing antibodies declined more slowly than total anti-RBD and anti-N IgG, and the two neutralizing-antibody clusters decayed even more slowly than total neutralizing antibodies. Interestingly, the level of neutralizing antibodies at 12 months after disease onset was significantly lower than that at 2 months but more broadly neutralized SARS-CoV-2 variants, including Alpha (B.1.1.7), Beta (B.1.351), Gamma (P.1), Delta (B.1.617.2), and Lambda (C.37). Significant immune escape by the Omicron variant (B.1.1.529) was also observed 2 months post-recovery. Furthermore, we revealed that a high percentage of virus-specific CD4^+^ T cells and cTfh1 were associated with a slower decline in humoral immunity, accompanied by higher levels of CXCR3 ligands such as CXCL9 and CXCL10, higher frequency of cTfh1, and lower levels of cTfh2 and cTfh17. Our data highlight the importance of coordinating T-cell and humoral immunity to achieve long-term protective immunity.

## Introduction

Since late 2020, dozens of COVID-19 vaccines have been developed, with efficacies varying from 50% to 92%.^1^ Several preliminary studies (Moderna and Pfizer-BioNTech) investigated the duration of vaccine effectiveness and suggested that a booster shot is needed 6–8 months after complete immunization.^2,3^ However, some important questions regarding why vaccine-induced protection wanes so quickly remain to be answered. Additionally, the duration and effectiveness of infection-induced immunity in patients who have recovered from SARS-CoV-2 infection and the factors that facilitate and maintain long-term memory immunity against SARS-CoV-2 are still unknown.

Growing evidence has shown that infection- or vaccination-induced long-term memory can be evaluated by quantifying SARS-CoV-2 circulating antibodies, memory B cells, CD8^+^ T cells, and CD4^+^ T cells. However, this quantification does not entirely represent the complete protective immunity. A few studies have provided contradictory reports on the duration of SARS-CoV-2 antibodies after SARS-CoV-2 infection, raising concerns about the short-lived protective immunity against COVID-19. Long et al.^4^ reported that SARS-CoV-2 antibodies rapidly wane as most study participants had detectable neutralizing responses lasting several months, and that asymptomatic patients had relatively low antibody responses, which disappeared quickly. Seydoux et al.^5^ sequenced circulating antibodies and showed that only a few somatic mutations in potent neutralizing antibodies occurred within a SARS-CoV-2-infected individual. In a parallel study, lyer et al.^6^ found that spike antibodies decline slower than N antibodies at approximately 200 days post-infection. Additionally, Piccoli et al.^7^ revealed that RBD-specific serum IgG titers had a half-life of 49 days, and nAb titers and avidity increased over time for some individuals, which is consistent with affinity maturation. Dan et al.^8^ demonstrated that spike IgG titers were durable, with modest declines in titers at 6 to 8 months after disease onset at the population level, and that anti-RBD IgG and SARS-CoV-2 nAb titers were potentially similarly stable, consistent with the RBD domain of spike being the dominant nAb target.

T-cell immunity plays an important role in the suppression of SARS-CoV-2 infection. Recent studies has shown that moderate and severe symptoms in patients with COVID-19 are associated with a drastic reduction in the numbers of both CD4^+^ and CD8^+^ T cells.^9,10^ We have recently demonstrated that a balance between T-cell immunity and neutralizing antibodies is required for COVID-19 recovery.^11^ Additionally, Son et al.^12^ uncovered the presence of a tissue-resident helper T-cell population in the lung, which plays a critical role in promoting the development of protective B and CD8^+^ T-cell responses. Goel et al.^13^ reported that mRNA vaccination further induced antigen-specific CD4^+^ and CD8^+^ T cells and that early CD4^+^ T-cell responses were correlated with long-term mRNA vaccine-induced humoral immunity. Kaneko et al.^14^ reported that patients with COVID-19 exhibit a loss of Bcl-6 expressing follicular helper T cells and germinal centers (GCs), suggesting a low chance for long-lived memory or high-affinity B plasma cells.

In this study, we evaluated the durability of various SARS-CoV-2-specific antibodies by comparing their titers, decay rates, and half-lives in the blood samples collected from individuals 2 and 12 months after COVID-19 onset, and further analyzed the relationship between the frequencies of CD4^+^ or CD8^+^ and the decay rates of SARS-CoV-2-specific antibodies. Based on these data, we aimed to determine the host factors that could influence the maintenance of long-term protective immunity.

## Results

### Neutralizing antibodies decay slower than binding antibodies

**Fig. 1:**
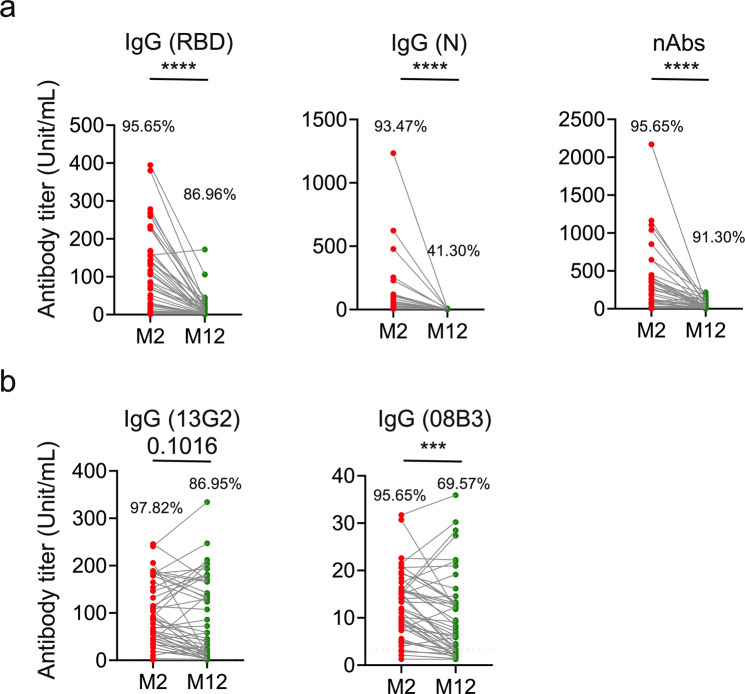
Neutralizing antibodies show a slower decay rate than binding antibodies. Two blood samples were collected from convalescent individuals with COVID-19 at approximately 2 (M2) and 12 months (M12) after disease onset (*n* = 46). The antibody levels at M2 and M12 were measured (*n* = 46). Total IgG to RBD and N in the plasma samples were examined using ELISA, and nAb levels were examined using the focus reduction neutralization test (FRNT_50_). **a** Pair comparisons of IgG to RBD, N, and nAbs between M2 and M12. **b** Pair comparisons of antibody levels to 13G2 and 08B3 clusters. Each dot represents an individual participant. ***p < 0.001 and ****p < 0.0001. Wilcoxon rank-sum test was used to compare paired continuous variables that were not normally distributed. The percentage above the dot plots indicates the detection rate of each group.

To assess the durability of SARS-CoV-2-specific antibodies, we measured the collective levels of anti-RBD IgG, anti-N IgG, and total neutralizing antibodies (nAbs). Blood samples were collected at 2 and 12 months (denoted as M2 and M12, respectively) after disease onset. As shown in Fig. 1a, the percentages of patients whose antibody titers reached levels higher than the cutoff value (RBD = 0.4729; N = 2.8103; nAb = 1) were 95.65% (M2) and 86.96% (M12) for IgG (RBD); 93.47% (M2) and 41.30% (M12) for IgG (N); and 95.65% (M2) and 91.30% (M12) for nAbs. The comparison of the data at M2 and M12 using a Wilcoxon rank-sum test showed a significant reduction in the levels of IgG (RBD), IgG (N), and total nAbs over 12-months (*p* < 0.0001).

We also estimated the average antibody decay rate by fitting the longitudinal data from the 46 patients to an exponential decay model using a Bayesian hierarchical framework (detailed in Materials and Methods). The key outputs of the model fitting including the distribution of the mean decay rate and the associated half-lives of the different antibody populations are listed in Table 1. The decay rate of anti-RBD IgG (median 0.249, 95% CI: 0.21–0.287), anti-N IgG (median 0.35, 95% CI: 0.298–0.414), and total nAbs (median 0.135, 95% CI: 0.089–0.188) are shown in Table 1.

**Table 1:** Estimates of decay rate and associated half-life for different antibodies and CD4^+^ T-cell levels

		Decay rate (per month)	Half-life (month)
		Median (95% CI)	Median (95% CI)
IgG (RBD)	Total (*n* = 45)	0.249 (0.21–0.287)	2.79 (2.42–3.3)
	CD4 ≤ 10% (*n* = 24)	0.265 (0.21–0.323)	2.62 (2.14–3.3)
	CD4 > 10% (*n* = 21)	0.229 (0.17–0.286)	3.03 (2.42–4.08)
IgG (N)	Total (*n* = 42)	0.35 (0.298–0.414)	1.98 (1.67–2.33)
	CD4 ≤ 10% (*n* = 19)	0.384 (0.233–0.788)	1.8 (0.88–2.97)
	CD4 > 10% (*n* = 21)	0.36 (0.305–0.428)	1.93 (1.62–2.27)
nAbs	Total (*n* = 44)	0.135 (0.089–0.188)	5.13 (3.68–7.79)
	CD4 ≤ 10% (*n* = 24)	0.15 (0.0843–0.238)	4.62 (2.91–8.22)
	CD4 > 10% (*n* = 20)	0.132 (0.0675–0.199)	5.27 (3.47–10.3)
IgG (13G2)	Total (*n* = 45)	0.0344 (0.0114–0.073)	20.2 (9.5–60.8)
	CD4 ≤ 10% (*n* = 24)	0.108 (0.0458–0.181)	6.4 (3.83–15.1)
	CD4 > 10% (*n* = 21)	0.00898 (0.000702–0.0312)	77.2 (22.2–987)
IgG (08B3)	Total (*n* = 42)	0.0369 (0.0149–0.0644)	18.8 (10.8–46.6)
	CD4 ≤ 10% (*n* = 21)	0.0836 (0.037–0.172)	8.29 (4.04–18.8)
	CD4 > 10% (*n* = 21)	0.0166 (0.00207–0.0385)	41.8 (18–335)

### Two neutralizing antibodies clusters, represented by clones 13G2 and 08B3, show minimal decay

To assess the wide range and durability of SARS-CoV-2-specific neutralizing antibodies, a competitive ELISA assay was established using two neutralizing monoclonal antibody clones, 13G2 and 08B3. These monoclonal antibodies were harvested from the ascites of mice injected with two hybridoma cell lines generated from mice immunized with the SARS-CoV-2 RBD. Clone 13G2 had been shown to neutralize both the wild-type SARS-CoV-2 and Beta variants, whereas clone 08B3 was shown to neutralize the wild-type but not Beta variants (unpublished data).

Competitive ELISA was performed to measure the levels of two nAb clusters targeted or shared with the epitopes of two nAb clones, 13G2 and 08B3, in sera of 46 individuals who had recovered from COVID-19. As shown in Fig. 1b, the percentages of patients whose antibody titers reached levels above the cutoff value were 97.82% (M2) and 86.95% (M12) for the 13G2 cluster, and 95.65% (M2) and 69.57% (M12) for the 08B3 cluster. Additionally, the M2 and M12 data were compared using the Wilcoxon rank-sum test. The results showed that 08B3 clusters had a significant reduction over 12 months (*p* < 0.0144); in contrast, there was no significant decline in the mean level of 13G2 clusters (*p* = 0.1333, Fig. 1b). The decay rate of 13G2 clusters was 0.0344 per month with a 95% CI of 0.0114–0.073, which is comparable to that of 08B3 clusters (median 0.0369, 95% CI: 0.0149–0.0644) but much lower than those of IgG (RBD), IgG (N), and the total nAbs (Table 1). The median estimates of the half-lives of 13G2 and 08B3 clusters are 18.8 and 20.2 months, respectively, which are 3–10x longer than those of the other antibody populations (Table 1), suggesting a more prominent role of these antibody clusters in protective host immune responses to SARS-CoV-2 infection.

### Broadly neutralizing antibodies against different SARS-CoV-2 variants persist one year after infection

The emergence of SARS-CoV-2 variants has raised concerns regarding the breadth of neutralizing-antibody responses. We compared the neutralizing-antibody levels in plasma at M2 and M12 in response to a control virus, WH-1 (wild-type SARS-CoV-2), and five variants including, B.1.1.7 (Alpha) identified from the United Kingdom, B.1.351 (Beta) from South Africa, P.1 (Gamma) from Brazil, and B.1.617.2 (Delta) from India, and C.37 (Lambda) from South America.

**Fig. 2:**
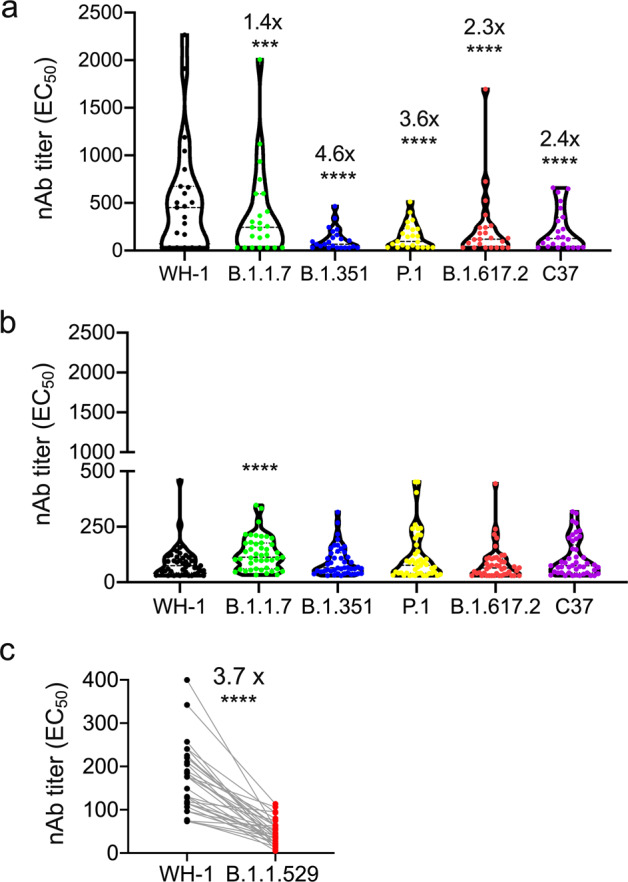
Broadly neutralizing antibodies against different SARS-CoV-2 strains 1 year after infection. Blood samples were collected from convalescent patients with COVID-19 at 2 (M2) and 12 months (M12) after disease onset (M2, *n* = 23; M12, *n* = 45). **a**, **b** Each dot represents an individual participant. Violin plot analysis comparing the distribution of nAb titers of M2 (**a**) and M12 (**b**) recovered plasma against WH-1 and VOCs, including B.1.1.7, B.1.351, P.1, B.1.617.2, and C.37, determined by using a pseudovirus-based neutralization assay. **c** The EC_50_ of neutralizing antibodies against WH-1 and B.1.1.529 (Omicron) was determined in the plasma of convalescence at M2 (*n* = 32). The fold change was expressed as the ratio of WH-1 to B.1.1.529. Each dot represents an individual participant. ***p < 0.001 and ****p < 0.0001. Wilcoxon rank-sum test was used to compare paired continuous variables that were not normally distributed

Plasma samples collected from 23 and 45 convalescent individuals at M2 and M12, respectively, were analyzed using a pseudovirus-based neutralization assay. The results show that an adequate amount of neutralizing antibodies against WH-1 was detected in the blood plasma at M2, while a significant decrease of neutralization activity against variants B.1.1.7, B.1.351, P.1, B.1.617.2, and C.37, with a respective fold reduction of 1.4, 4.6, 3.6, 2.3, and 2.4 was observed (Table 2 and Fig. 2a). Convalescent plasma at M12 showed 3.3-fold reduced geometric mean (GM) neutralization EC_50_ against WH-1 (Table 2), which indicates a significant antibody decline. However, there was no reduction of neutralization activity against the variants at M12 compared to wild-type virus. Interestingly, the geometric mean EC_50_ of the nAb titers (EC_50_ GMTs) against the variants at M2 and M12 was largely the same, except for a slight decrease for B.1.617.2 (*p* = 0.0423, Table 2). The nAb titers of B.1.1.7, B.1.351, P.1, B.1.617.2, and C.37 variants were 1.4-, 4.6-, 3.6-, 2.3-, and 2.4-fold lower than that of WH-1 at M2, respectively; however, there was no significant reduction in the level of nAbs against variants compared to that against the wild-type at M12. Furthermore, a considerable number of nAbs (average 64.6-109.5, Table 2) were detected at M12, indicating that protective immunity was maintained at a certain level over a year. Interestingly, only marginal differences were observed in the cross-reactivity between the wild-type and different strains at M12 (Fig. 2a), indicating that the remaining nAbs may have been selected and broadly targeted different strains with higher affinity. Therefore, we speculate that relatively high-affinity nAbs will be competitively selected towards lower amount of remaining nAbs at 12 months but with more cross-reactivity between different SARS-CoV-2 strains.

**Table 2:** Neutralizing-antibody response to WH-1 and 5 variants at 2-months (M2) and 12-months (M12)

	M2 (*n* = 23)	M12 (*n* = 45)	*p*-value
Age	48.3 (43.50–53.08)	49.2 (45.20–53.10)	0.7883
Female	30.4	48.9	0.507
EC_50_ GMT (95% CI)			
WH-1	237.9 (123.0–460.2)	72.3 (59.6–131.6)	0.0044
B.1.1.7	179.7 (99.35–325.1)	109.5 (91.2–131.6)	0.0652
B.1.351	78.8 (53.8–115.3)	76.3 (63.15–92.1)	0.8167
P.1	96.7 (63.35–147.6)	80.6 (62.9–103.2)	0.6725
B.1.617	116.6 (70.6–192.5)	64.6 (52.77–79.1)	0.0423
C.37	122.5 (75.1–199.8)	82.7 (66.2–103.2)	0.2513

Data are means (age), number of participants (%, female) or geometric mean titers (GMT). Comparisons of numerical variables and categorical variables from complex sample surveys were performed using the Mann–Whitney test and the chi-squared test of Fisher’s exact test, respectively.

To examine the neutralization sensitivity of the Omicron variant compared to the wild-type SARS-CoV-2, B.1.1.529 (Omicron) was tested against blood plasma of 41 convalescent patients with COVID-19 who were infected with WH-1. The results showed that for patients who recovered from WH-1, the EC_50_ GMTs of the ancestral virus (GM 177.6, 95% CI: 145.3–210.0) was 3.7-fold (individual fold change ranging from 1.19 to 25.54) lower than that of B.1.1.529 (GM 48.06, 95% CI: 37.79–58.32). These data indicate that the Omicron variant escaped the wild-type-specific nAbs, suggesting a significant immune escape (Fig. 2c).

### High levels of virus-specific CD4^+^ T cells at the early convalescent phase correlate with long-term nAb levels

**Fig. 3:**
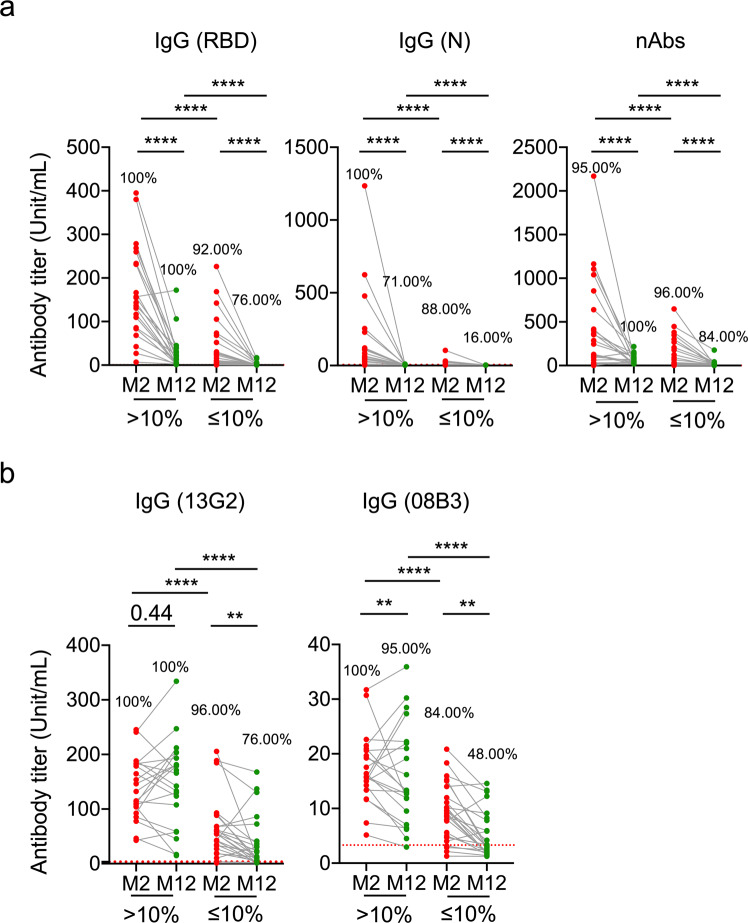
High levels of virus-specific CD4^+^ T cell at early convalescent phase correlate with long-term maintenance neutralizing antibodies. Antibodies present in plasma of the high- (*n* = 23) and low-CD4^+^ groups (*n* = 23) against RBD and N, and nAbs in the plasma at M2 and M12, were examined using ELISA and FRNT_50_ assays. **a**, **b** Pair comparisons of IgG levels against RBD and N and nAbs between M2 and M12 for the high-and low-CD4 + groups; **b** Pair comparisons of IgG levels against the 13G2 and 08B3 clusters. Percentages on top of dot plots represent frequencies of samples with antibody titers above the cutoff. Each dot represents an individual participant. **p < 0.01 and ****p < 0.0001. Student’s *t*-test was used to analyze differences in the mean values between groups. The Mann–Whitney test was used to compare the central tendencies of the two groups (mean or median). Wilcoxon rank-sum test was used to compare paired continuous variables that were not normally distributed. The percentage above the dot plots indicates the detection rate of each group.

To determine whether CD4^+^ T cells regulate long-term humoral immunity in patients with COVID-19, we compared the estimated decay rates for 10 months between two groups of patients grouped by the frequency of IFNγ^+^TNF^+^CD4^+^ T cells responding to SARS-CoV-2 peptide pool stimulation followed by 10-day expansion: high-CD4^+^ group (frequency > 10%) and low-CD4^+^ group (frequency ≤ 10%). The estimated mean decay rates in the low-CD4^+^ group were higher than those in the high-CD4^+^ group (Fig. 3a, b and Table 1), implying that the decay rates were negatively associated with the frequency of virus-specific CD4^+^ T cells measured at M2. Notably, for the 13G2 clusters, the estimated median decay rate for the low-CD4^+^ group was 12-fold higher than that in the high-CD4^+^ group (decay rate: 0.108 vs. 0.00898; half-life: 6.4 vs. 77.2 months; Table 1). Similarly, for the 08B3 cluster, there was an approximately 5-fold difference between the low-and high-CD4^+^ groups (decay rate: 0.0836 vs. 0.0166; half-life: 8.29 vs. 41.8 months; Table 1). Additionally, we observed moderate (or less) but significant differences in the estimated mean decay rates between the low- and high-CD4^+^ groups (Table 1) for anti-RBD-IgG (decay rate: 0.265 vs. 0.229; half-life: 2.62 vs. 3.03 months), anti-N IgG (decay rate: 0.384 vs. 0.36; half-life: 1.8 vs. 1.93 months), and total nAbs (decay rate: 0.15 vs. 0.132; half-life: 4.62 vs. 5.27 months). Furthermore, our data also showed virus-specific CD4^+^ T cells at 2 months (Supplementary Fig. 1a, b) and 12 months (Supplementary Fig. 2a, b) are correlated with nAbs respectively, not only in total antibody level (Supplementary Figs. 1a and 2a), but also in nAb clones 13G2 and 08B3 level (Supplementary Figs. 1b and 2b).

### The level of CXCR3 ligands correlates with the percentage of virus-specific CD4^+^ T cells

**Fig. 4:**
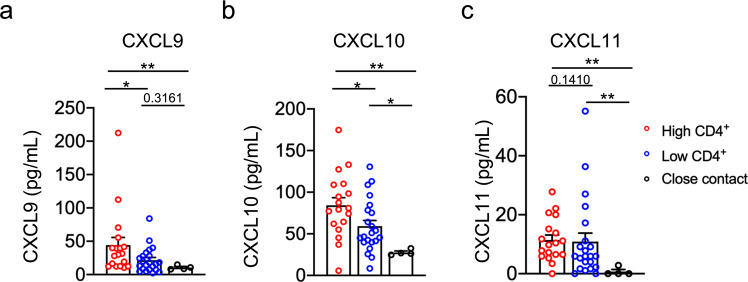
Peripheral chemokines related to CD4^+^ T cells in convalescent patients with COVID-19. The levels of CXCL9, CXCL10, and CXCL11 were determined for high- (*n* = 18) and low-CD4^+^ (*n* = 22) convalescent individuals with COVID-19 and close contacts (*n* = 4) at M2 using cytometric Beads array. **a**–**c** Dot plot analysis of (**a**) CXCL9, (**b**) CXCL10, and (**c**) CXCL11 levels among high-convalescent and low-CD4^+^convalescent patients with COVID-19, and close contacts, respectively. Each dot represents an individual participant. Bars represent mean values. *p < 0.05 and **p < 0.01. Student’s *t*-test was used to analyze differences in the mean values between groups. The Mann–Whitney test was used to compare the central tendencies of the two groups (mean or median). Data are presented as mean ± SEM.

The CXCR3 receptor reacts with three interferon-inducible chemokines, CXCL9, CXCL10, and CXCL11, which are crucial for directing activated T cells to the sites of inflammation. These chemokines have been implicated in the induction and perpetuation of several viral infections by mediating CD4^+^ T-cell recruitment, activation, and function.^15–17^ Given that recruitment of CD4^+^ T cells to infected areas may be one of the mechanisms that account for virus-specific CD4^+^ maintenance, we investigated the expression of SAR-CoV-2 infection-induced CXCR3 ligands CXCL9, CXCL10, and CXCL11. Using the CBA assay, we detected the plasma levels of CXCL9, CXCL10, and CXCL11 at M2. The results showed that plasma CXCL9 and CXCL10 levels were higher in the high-CD4^+^ group than in both low-CD4^+^ and close contact groups at M2, while there was no difference in plasma CXCL11 levels between the high- and low-CD4^+^ groups (Fig. 4).

**Fig. 5:**
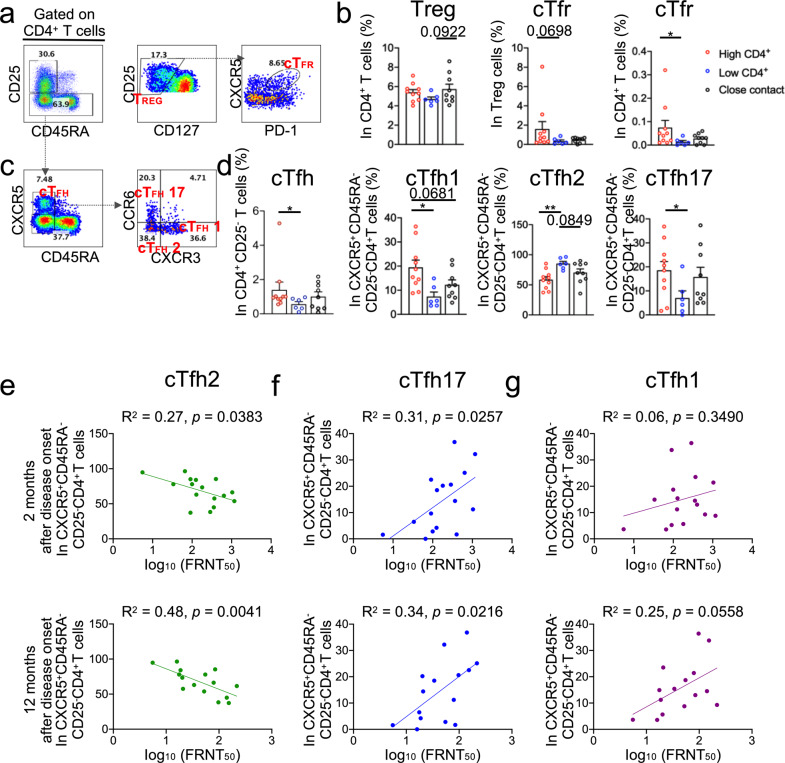
Peripheral CD4^+^ T-cell subsets in convalescent patients with COVID-19. T-cell subsets were analyzed by fluorescence-activated cell sorting (FACS) in peripheral blood mononuclear cell (PBMCs) from high- (*n* = 10) and low-CD4^+^ (*n* = 6) convalescent individuals with COVID-19 and close contacts (*n* = 9). **a** Gating strategies for Treg cells (CD25^+^CD45RA^−^CD127^−^) and cTfr cells (CD25^+^CD45RA^–^CD127^–^CXCR5^hi^PD-1^hi^). **b** Dot plot analysis of the frequencies of Treg and cTfr cells in high- and low-CD4^+^ convalescent individuals with COVID-19 and close contacts. **c** Gating strategies for different peripheral circulating Tfh cell subsets, including CXCR3^+^CCR6^−^cTfh (cTfh1) cells, CXCR3^–^CCR6^–^ cTfh (cTfh2) cells, and CXCR3^–^CCR6^+^ cTfh (cTfh17) cells. **d** Frequencies of cTfh1, cTfh2, and cTfh17 cells in high-and low-CD4^+^ COVID-19 convalescent individuals with COVID-19 and close contacts. **e**–**g** Correlation analysis of (**e**) cTfh2, (**f**) cTfh17, and (**g**) cTfh1 cells with SARS-CoV-2-specific neutralizing-antibody titers in high-and-low-CD4^+^ convalescent individuals with COVID-19 (*n* = 16). Each dot represents an individual participant. Bars represent mean values. *p < 0.05 and **p < 0.01. Student’s *t*-test was used to analyze differences in the mean values between groups. The Mann–Whitney test was used to compare the central tendencies of the two groups (mean or median). Data are presented as mean ± SEM.

Furthermore, the frequencies of cTfh1 and cTfh17 cells were reported to correlate with the neutralizing activity measured in the serum in response to vaccines or during viral infection.^18,19^ Therefore, we further measured the frequency of various T-cell subsets, including cTfh (cTfh1, cTfh2, and cTfh17) and Tregs at M2. There was a significant difference in the frequency of cTfh1 between the high- and low-CD4^+^ groups (Fig. 5d). We further examined the correlation between the frequency of cTfh1, cTfh2, cTfh17, and nAbs. Surprisingly, nAbs were negatively correlated with cTfh2 (Fig. 5e) but positively correlated with cTfh17 (Fig. 5f). There was a positive correlation between cTfh1 cells and nAbs at M12 (*R*^2^ = 0.2534, *p* = 0.0558; Fig. 5g); however, it was not statistically significant. Interestingly, the frequencies of cTfh1 were positively correlated to the M12/M2 ratios, representing the maintenance of antibodies, of both neutralizing antibodies 13G2 and 08B3 (Supplementary Fig. 3), which further supports our hypothesis that host humoral immunity continues to evolve after viral resolution to achieve affinity maturation at some individual neutralizing-antibody level. It is worth mentioning that there was a negligible difference in the frequency of peripheral Treg cells, but a significantly higher frequency of circulating Tfr (cTfr) cells (CD45RA^−^CD127^−^CD25^+^CXCR5^hi^PD-1^hi^) in higher virus-specific CD4^+^ group than in lower CD4^+^ group (Fig. 5b).

## Discussion

Determining the longevity of SARS-CoV-2 nAbs and understanding how they are sustained is important for designing and assessing vaccination strategies to combat the COVID-19 pandemic. Here, we report that SARS-CoV-2 nAbs can be sustained for at least one year in convalescent individuals who have recovered from COVID-19. Although a significant decline in anti-RBD IgG, anti-N IgG, and total nAbs was detected, the levels of the two selected nAb clusters were steadily maintained over 12 months. The total nAbs waned more slowly than the total anti-RBD IgG and total anti-N IgG, indicating that host humoral immunity possibly continues to evolve after viral resolution to achieve affinity maturation in GC through mutations in ancestral sequences of antibodies. This assumption is consistent with a recent finding that spike-specific memory B cells were more abundant at 6 months than at 1 month after symptom onset.^8^ Furthermore, the half-lives of two neutralizing-antibody clusters (represented by clone 13G2 and clone 08B3) were considerably longer than those of the collective antibodies (total anti-RBD, anti-N, and total nAbs), and broad nAbs were more selected out at 12 months than at 2 months after disease onset. Our findings indicated that the decline in antibodies is influenced by antigen and antibody characteristics, and confirmed that the host immune system constantly endeavors to create heterogeneity in the antibody repertoire and select high-affinity nAbs, which are more specific and effective in protecting against infections.

SARS-CoV-2 variants continue to emerge, and it is unknown whether natural infection-acquired immunity against the wild-type virus will protect against variants at a certain time after disease onset. The duration of immunity resulting from natural infections, although not well understood, is suspected to persist for ≥90 days in most individuals.^20^ A model predicted that the average reinfection risk rises from approximately 5% at 4 months after the initial infection to 50% by 17 months.^21^ Our data suggest that protective immunity can be sustained for 12 months after infection but at a lower level than that 2 months after disease onset. Additionally, the nAbs at 12 months diminished the difference in cross-reactive neutralization against different strains observed at 2 months, indicating that the remaining nAbs were selected and were broader. This is also supported by the increasing level of the nAb clones 13G2 and 08B3 at 12 months in some patients compared to that at 2 months.

Many studies have suggested that various vaccine-induced anti-SARS-CoV-2 immunities only last between 6 and 12 months.^20,22–24^ Therefore, a third dose of vaccine was recommended for boosting immunity, indicating that current commercially available vaccines fail to induce long-term nAbs. In contrast, a previous study suggested that patients who have recovered from COVID-19 have a lower risk of reinfection, suggesting that natural immunity against SARS-CoV-2 confers a neutralizing capacity for at least a year.^25^ Therefore, exploring the differences in the vaccine- and infection-induced protective immunity will help reveal the factors that play a role in maintaining long-term neutralizing immunity. The present study revealed that slow antibody decay rates are associated with a higher frequency of virus-specific CD4^+^ T cells, indicating the importance of cellular and humoral immunity coordination in maintaining long-term protective immunity. It also is possible that the recruitment and trafficking of virus-specific T cells were also mediated by high levels of CXCR3 ligands, such as CXCL9 and CXCL10.

Circulating Tfh1 cells represent germinal center Tfh cells and have an important role in T-cell-dependent B-cell maturation and antibody production.^26^ Some recent studies show that activation of cTfh1 cells is associated with different forms of humoral immunity (antibody and/or antibody-secreting cells and/or antigen-specific B cells) for a number of viral infections and vaccines, including influenza,^27–29^ HIV,^30^ and HCV.^31^ These studies found that the population of cTfh cells in peripheral blood of vaccinated or infected individuals are strongly associated with the development of protective humoral immunity. Consistent with a recent report that the cTfh subsets are informative biomarkers of the level of neutralizing antibodies of SARS-CoV-2,^32^ our data here show that the numbers cTfh1 in the blood plasma are correlated to the maintenance of some specific clusters of neutralizing antibodies. Furthermore, higher cTfr in higher CD4^+^ group indicated that GC response in higher CD4^+^ group was under selective pressure on the most competitive B-cell clones, thereby enhancing the high-affinity response.^33^

In this study, we did not perform in vivo studies on B maturation in the germinal center of lymph node, our finding concerning the increase of individual neutralizing antibody from 2 months to 12 months is only indirect proof for antibody maturation. Albeit of above limitations, our findings still give the hint that future COVID-19 vaccines need to consider CD4^+^ T cells, cTfh1 and antibodies to achieve long-term protective immunity.

## Materials and methods

### Cohort and sample preparation

In this study, 46 patients with COVID-19 (antibody-positive and/or nucleic acid test positive, Ab^+^ and/or NAT^+^) and 39 previously defined close contacts (Ab^-^ and NAT^-^) were recruited and provided signed informed consent (supplementary Table 1). As we have previously described,^34^ close contacts were determined as family members or friends who had stayed with an individual(s) infected with SARS-CoV-2 5 days before disease onset or hospitalization. Two blood samples were collected from each patient at around 2 months (M2: ranging from 48 to 86 days) and 12 months (M12: ranged from 363 to 390 days) after disease onset. The medical data collected from the individuals with COVID-19 included symptoms at disease onset and records of physical examinations, laboratory tests, and tomographic imaging. This study was approved by the Ethics Commission of the First Affiliated Hospital of Guangzhou Medical University (No.2020-51). The serum and peripheral blood mononuclear cells (PBMCs) were prepared as previously described.^34^

### Detection of blood plasma IgG in convalescent individuals with COVID-19

SARS-CoV-2-specific IgG in the blood plasma was detected separately using two ELISA kits targeting the N protein and S-RBD (Guangzhou DARUI Co., Ltd, China). The cutoff value for the assignment of positive samples was determined according to the manufacturer’s instructions. To standardize the assay and quantify the results, an arbitrary titer (U/mL) was assigned to each tested sample based on the standard curve drawn from a dilution series of a sample collected from a patient with COVID-19 and a high level of IgG (anti-RBD, anti-N).

### Focus reduction neutralization test (FRNT)

The SARS-CoV-2 FRNT was performed in a certified BSL-3 laboratory. Fifty microliters of plasma samples were serially diluted, mixed with 50 μL of SARS-CoV-2 (100 focus forming units [FFU]) in 96-well microwell plates, and incubated for 1 h at 37 °C. Mixtures were then transferred to 96-well plates seeded with Vero E6 cells (ATCC, USA) and incubated for 1 h at 37 °C to allow virus entry. The inocula were removed before adding overlay media (100 μL MEM containing 1.2% carboxymethylcellulose). The plates were then incubated at 37 °C for 24 h. The overlays were removed, and the cells were fixed with a 4% paraformaldehyde solution for 30 min. The cells were permeabilized with 0.2% Triton X-100 and incubated with cross-reactive rabbit anti-SARS-CoV-2 N IgG (Cat #40143-R001; Sino Biological Inc, China.) for 1 h at room temperature before adding HRP-conjugated goat anti-rabbit IgG (H + L) antibody (1:4000 dilution; Cat # 111-035-144; Jackson ImmunoResearch Laboratories Inc., USA). The cells were incubated at room temperature. The reactions were developed using KPL TrueBlue peroxidase substrates (Seracare Life Sciences Inc., USA). The number of SARS-CoV-2 foci was calculated using an EliSpot reader (Cellular Technology Ltd., USA).

### Competitive ELISA (cELISA) for the detection of neutralizing-antibody clusters

The nAb clones 13G2 and 08B3 were purified using a HiTrap™ Protein G column (GE Healthcare, USA), followed by conjugation with horseradish peroxidase (HRP) using EZ-Link™ Plus Activated Peroxidase (Thermo Fisher Scientific, USA), according to the manufacturer’s instructions. HRP-13G2 or HRP-08B3 was dialyzed with Slide-A-Lyzer Dialysis Cassettes (Thermo Fisher Scientific, USA) against PBS and stored in a Pierce™ Peroxidase Conjugate Stabilizer (Thermo Fisher Scientific, USA). The established cELISA was performed in a Corning® 96-Well Clear Flat Bottom Polystyrene High Bind Microplate (Corning Inc., USA). Briefly, plates were coated overnight with SARS-CoV-2 Spike/RBD protein (1 μg/mL, 100 μL/well) in PBS (without calcium and magnesium, pH 7.4; Thermo Fisher Scientific, USA) at 4 °C. After washing thrice with PBST, the plates were blocked with blocking buffer and incubated at 37 °C for 1 h. Then, 50 μL of diluted serum samples were added to each well and mixed well by pipetting. The plates were then incubated at 37 °C for 1 h. After washing thrice, 100 μL of diluted HRP-13G2 or HRP-08B3 was added and incubated at 37 °C for 30 min. After washing five times, 100 μL of room-temperature TMB Stabilized Chromogen (Invitrogen, Waltham, MA, USA) was added and incubated at 37 °C for 10 min, after which 50 μL/well of stop solution 2 N sulfuric acid (R & D Systems, USA) was added. The absorbance was measured at 450 nm using a Multiskan™ GO Microplate Spectrophotometer (Thermo Fisher Scientific, USA). The OD450 of the samples was converted to a percent inhibition (PI) value using the following formula: PI (%) = (OD450 value of negative controls – OD450 value of sample)/OD450 value of negative controls × 100%, and the inhibition rate >30% were determined as positive read for nAb by using 88 acute COVID-19 samples in another study (not shown here). Furthermore, to standardize the assay and quantify the results, an arbitrary titer (U/mL) was given to each tested sample based on the standard curve drawn from a dilution series of a sample collected from a patient with COVID-10 and a high IgG level (anti-13G2 and anti-08B3).

### Pseudovirus-based neutralization assay

SARS-CoV-2 and six variants were examined and chosen to represent the original SARS-CoV-2 strain and emerging variants with mutations in the spike protein. Neutralization was measured by reducing the *luc* gene expression, as previously described for the HIV pseudovirus neutralization assay.^35^ The 50% inhibitory dilution (EC_50_) was defined as the serum dilution at which the relative light units (RLUs) were reduced by 50% compared to the virus control wells (virus + cells) after subtraction of the background RLUs in the control groups with cells only. Briefly, the pseudovirus was incubated with serial dilutions of the test samples (six dilutions in a 3-fold stepwise manner) in duplicate for 1 h at 37 °C, together with the virus and cell control wells in hexaplicate. Freshly trypsinized cells were added to each well. Following 24 h of incubation at 5% CO_2_ and 37 °C, luminescence was measured, as described in the section for pseudovirus titration. EC_50_ values were calculated with non-linear regression, that is, log (inhibitor) vs. response (four parameters), using GraphPad Prism 8 (GraphPad Software, USA).

### PBMC peptide stimulation and in vitro expansion

As previously described,^34^ SARS-CoV-2-specific peptides were designed and synthesized. For in vitro culture and stimulation, 1 × 10^6^ PBMCs were treated with the peptide pool (125 nM/peptide) and incubated for 10 days. During this culture, half of the medium was changed twice per week with fresh PRMI 1640 supplemented with 10% heat-inactivated FBS (Biological Industries, Israel), 100 U/mL penicillin (Gibco, Thermo Fisher Scientific, USA), and 0.1 mg/mL streptomycin (Gibco, Thermo Fisher Scientific, USA), and 10 U/mL rIL-2. The cells were then re-stimulated on day 10 with a medium containing the peptide pool (125 nM/peptide) overnight before staining for fluorescence-activated cell sorting (FACS) analysis. Virus-specific T cells (CTL and virus-specific CD4^+^ T cells) were detected as described.^34^

### Detection of T-cell subsets (Tfh, cTfh1, cTfh2, cTfh17, Tfr, and Treg)

Before antibody staining, frozen PBMCs were thawed and washed carefully. PBMCs (1 × 10^6^) were cultured in RPMI 1640 medium (Gibco, Thermo Fisher Scientific, USA) supplemented with 10% heat-inactivated FBS (Biological Industries, Israel), 100 U/mL penicillin (Gibco, USA), and 0.1 mg/mL streptomycin (Gibco, USA). Fc receptor blocking antibodies (BD Biosciences, USA) was used to block nonspecific staining of human lymphocytes for 15 min on ice.

For surface staining, cells were washed once with FACs buffer and incubated for 30 min at 25 °C in the dark with the following monoclonal antibodies at predetermined optimal dilutionsanti-CD3-AF700 (1:200, clone UCHT1, Cat# 557943; BD Biosciences, USA), anti-CD8-BV605 (1:200, clone RPA-T8, cat# 301039; BioLegend, USA), anti-CD4-PerCP Cy5.5 (1:100, Clone RPA-T4, cat# 560650; BD Biosciences), anti-CXCR5-BV421 (1:50, Clone RF8B2, cat# 562747; BD Biosciences), anti-CD25-BB515 (1:100, Clone 2A3, cat# 564467; BD Biosciences), anti-CXCR3-PE (1:50, Clone CEW33D, cat# 12-1839-42; eBioscience, Thermo Fisher Scientific, USA), anti-CD45RA-APC-H7 (1:100, clone HI100, cat# 560674; BD Biosciences), anti-CD127-BV786 (1:100, clone HIL-7R-M21, cat# 563324; BD Biosciences), anti-CCR6-PE-CY7 (1:100, clone G034E3, cat# 353317; BioLegend), and anti-PD-1-BV711 (1:100, clone EH12.2H7, cat# 329928; BioLegend). Live/dead aqua V510 was used to exclude dead cells. Following surface staining, the cells were washed twice with FACs buffer and kept at 4 °C throughout the acquisition using a BD LSRFortessa™ X-20 flow cytometer (BD Biosciences).

### Cytometric bead array (CBA)

CXCR3 ligands were measured using a cytometric bead array (CBA Human Soluble Protein Flex Set System, BD Biosciences, USA) on a BD FACSVerse flow cytometer (BD Biosciences, USA). A CBA was performed to detect the levels of CXCL9, CXCL10, and CXCL11. The experiments were conducted according to the manufacturer’s instructions, analyte signal intensities were calculated with reference to the respective standards, and absolute concentrations of individual analytes were calculated using BD FCAP Array Software (BD Biosciences, USA).

### Mathematical model and methods for the estimation of the antibody decay rate

The mathematical model that fit the antibody data was an exponential decay model:

$${{A}}\left( {12} \right) = {{A}}\left( 2 \right){{e}}^{ - 10{\lambda}},$$

where *A*(2) is the antibody measurements at the 2nd month and *A*(12) is the model-predicted antibody value at the 12th month lambda is the antibody decay rate (units per month).

We fit the model to the data using a Bayesian hierarchical modeling method, which is a well-established method for estimating biological parameters and has been used in many quantitative studies.^36–39^ Briefly, Bayesian inference is a statistical method to update our existing knowledge about the distribution of the biological parameter of interest (referred to as the prior distribution) by integrating the information from a set of novel data (by a likelihood function). The updated distribution of the parameter is called the posterior distribution, which is what we would like to obtain to provide an estimate for the parameter of interest θ. As the data points are grouped by patients, a hierarchical model is proposed by assigning the model to each patient (such that each patient will have a decay rate, which is called the individual parameter) and assuming that the decay rate for individuals follows a normal distribution characterized by a mean parameter (referred to as the population mean decay rate) and a between-subject variance parameter. We estimated the posterior distribution of the parameters in the hierarchical model by sampling the posterior distribution using the Hamiltonian Monte Carlo method, which was implemented in R (version 4.0.2) and Stan (version 2.21.0).^40^ The M3 method was used to deal with any censored data that were below the detection limit in the 12th month.^41^ Computer code is publicly available at (to be provided).

The estimated population mean decay rate was reported as the median estimate and 95% CI, which are given by the median value and the 2.5%–97.5% inter-quantile range of the marginal posterior distribution of the population mean decay rate, respectively. To estimate the half-life ${{t}}_{1/2}$, we first converted all posterior samples of the population mean decay rate to the half-life value using the non-linear transform ${{t}}_{1/2} = {{{\mathrm{log}}}}\left( 2 \right)/{\lambda}$ and then calculated the median and 95% CI.

### Statistical analyses

All statistical analyses were performed using GraphPad Prism software. Statistical significance was set at *p* < 0.05. *p < 0.05; **p < 0.01; ***p < 0.001, and ****p < 0.0001. Student’s *t*-test was used to analyze differences in the mean values between groups. The Mann–Whitney test was used to compare the central tendencies of the two groups (mean or median). Antibody responses were reported as geometric mean titers (GMT) with 95% (CI). Wilcoxon rank-sum test was used to compare paired continuous variables that were not normally distributed. The *χ*^2^ test or Fisher’s exact test was used to analyze categorical data. Cutoff values were assigned to evaluate the significance of the *p*-value according to the different statistical analysis methods indicated in each figure legend. All values are presented as mean ± SEM.

## Supplementary information

SUPPLEMENTAL MATERIAL

## Data Availability

All data generated or analyzed during this study are included in this published article and its supplementary information files.
