# Supplementary material for: SARS-CoV-2-specific CD4+ T cells are associated with long-term persistence of neutralizing antibodies
Source: Signal Transduct Target Ther. 2022 Apr 23;7:132. doi: 10.1038/s41392-022-00978-0 (PMC9034077; doi:10.1038/s41392-022-00978-0)
Supplement: Supplementary file 1 — SUPPLEMENTAL MATERIAL [file 41392_2022_978_MOESM1_ESM.pdf]

Supplementary Materials for

SARS-CoV-2-specific CD4+ T cells are associated with long-term persistence of neutralizing antibodies

Zhongfang Wang, Xiaoyun Yang, Xinyue Mei, Yumin Zhou, Zhiqiang Tang, Guichang Li, Jiaying Zhong, Mengqiu Yu, Mingzhu Huang, Xiaoling Su1, Bijia Lin, Pengxing Cao, Ji Yang, Pixin Ran
Correspondence to: pxran@gzhmu.edu.cn

This PDF file includes:

Figure. S1 to S3
Table S1

Supplementary Fig. 1

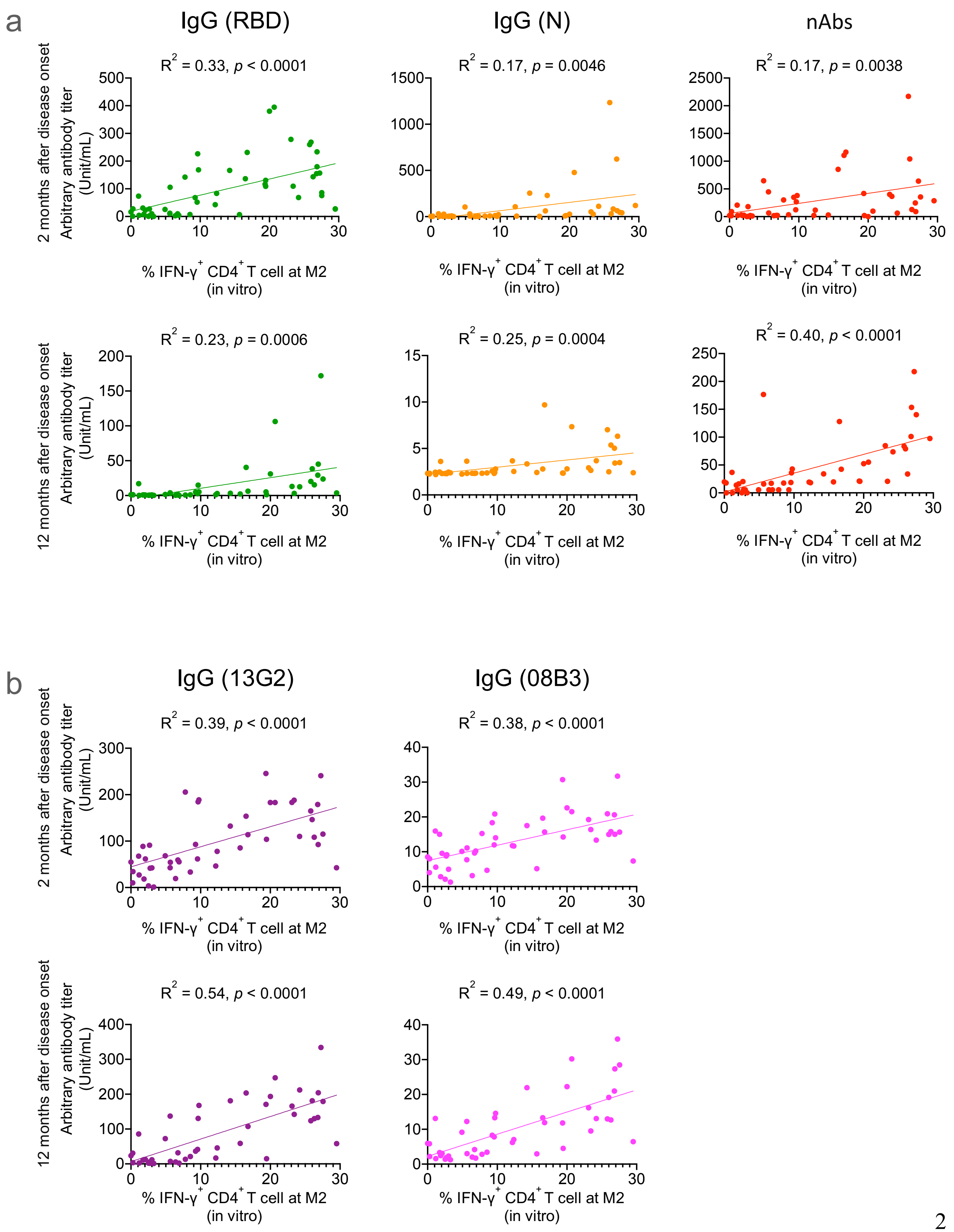

Figure. S1.

Virus-specific CD4$^+$ T cells at M2 correlate with a higher titer of neutralizing antibodies.

Correlation analysis on Virus-specific CD4$^+$ T cells with SARS-CoV-2-specific antibody titers. **a** The correlation analysis of virus specific CD4+ T cells at M2 with IgG (RBD), IgG(N) and nAb levels at M2 (upper panel, n = 46) and at M12 (lower panel, n = 46). **b** The correlation the frequencies of virus specific CD4+ T cells at M2 with IgG(13G2) and IgG(08B3) levels at M2 (upper panel, n = 46) and at M12 (lower panel, n = 46). Each dot represents an individual subject.

Supplementary Fig. 2

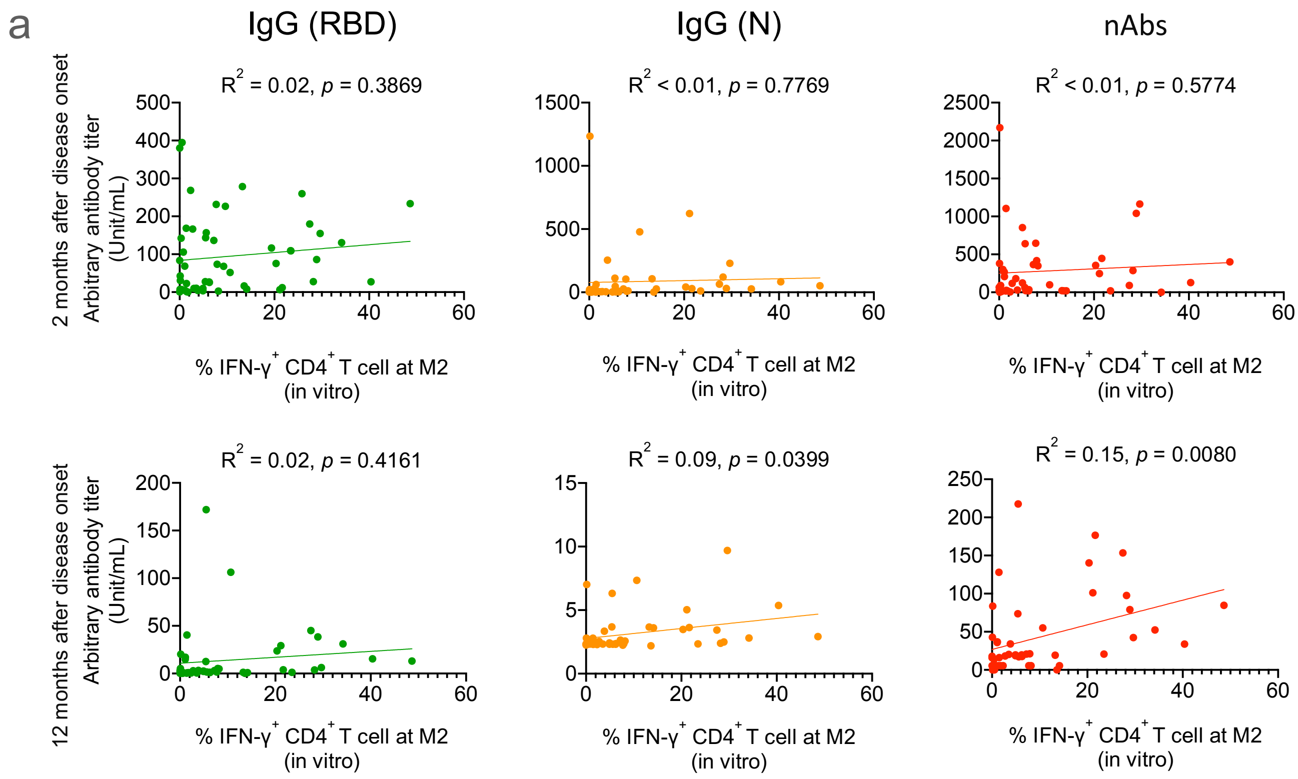

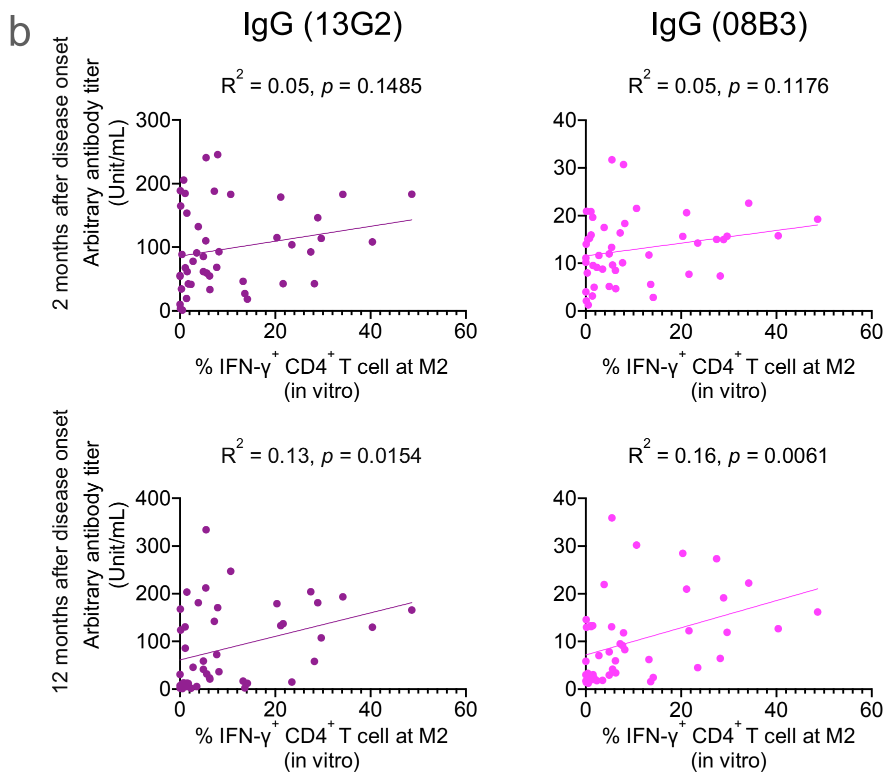

Figure. S2.

Supplementary Figure 2 Virus-specific CD4+ T cells at M12 were not associated with the antibody levels.

Correlation analysis of virus-specific $CD4^+$ T cells at M12 with SARS-CoV-2-specific antibody titers. **a** The correlation the virus specific CD4+ T cell frequencies at M2 with the levels of IgG (RBD), IgG(N) and nAb at M2 (upper panel n = 46) and at M12 (lower panel, n = 46). **b** The correlation of the frequencies of virus specific CD4+ T cells at M12 with antibody titers of IgG(13G2) and IgG(08B3) at M2 (upper panel, n = 46) and at M12 (lower panel, n = 46). Each dot represents an individual subject.

Supplementary Fig. 3

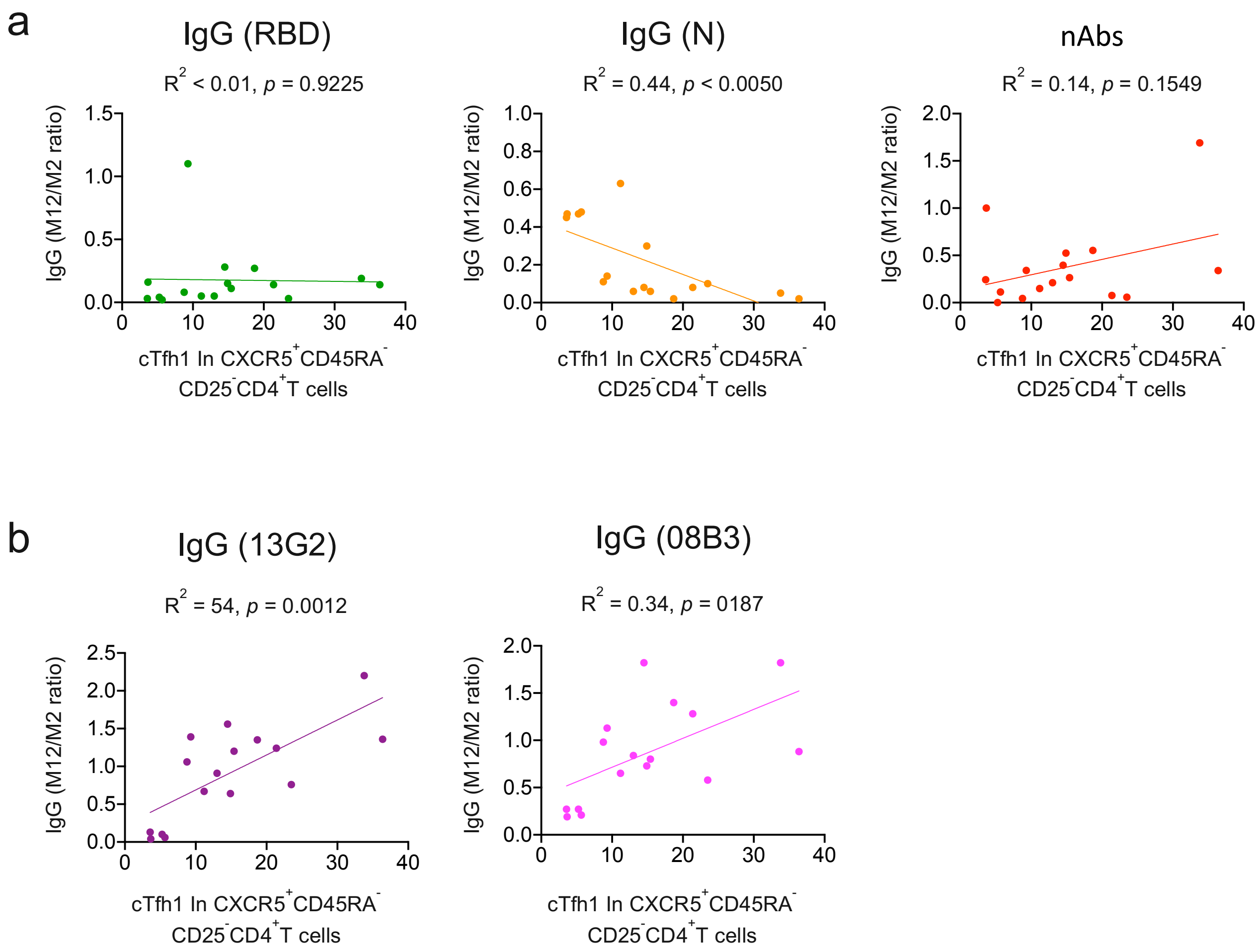

Figure. S3.

Supplementary Figure 3 13G2 cluster IgG maintenance was correlated to the level of cTfh1.

Correlation of antibody maintenance to cTfh1 cells. **a** Correlation analysis of cTfh1 cells to the waning rate (M12/M2 ratio) of anti-RBD, N IgGs and nAbs. **b** Correlation analysis of cTfh1 cells to the waning rate (M12/M2 ratio) of IgG (13G2) and IgG(08B3).

Table S1.

Supplementary Table 1: Demographics of 46 recovered COVID-19 patients

Category		COVID-19 (n=46)	Close contact (n = 36)
Age		49.2 (45.20 - 53.10)	39.7 (33.5 - 45.9)
Days after disease onset			
	2 months	51 (44 - 57)	NA
	12 months	377 (375 - 379)	NA
Gender			
	Female	23 (50%)	15 (42%)
	Male	23 (50%)	21 (58%)
Symptom			
	Severe ARDS	1 (3%)	NA
	Moderate	37 (80%)	NA
	Asymptomatic	8 (17%)	NA
Vaccination rates		0 %	0 %
